# Brachial plexus injury - from double to triple FFMT

**DOI:** 10.1186/1753-6561-9-S3-A29

**Published:** 2015-05-19

**Authors:** Yuan-Kun Tu

**Affiliations:** 1 E-Da Hospital, Kaohsiung, 82445 Taiwan

## Introduction

The concept of double free functioning muscle transfer (DFFMT) reconstruction for total arm type brachial plexus injury (BPI) had been successfully reported by Professor Doi from Japan. However, to overcome the weakness of finger/wrist extension after serving two functions by one gracilis muscle as described by Doi, we invented a new method of triple FFMT. This study was aimed to evaluate the clinical effectiveness of triple FFMT for total arm type BPI reconstruction.

## Methods

From 2001 to 2010, 85 patients received FFMT for total arm type BPI. Five patients were excluded from this study due to inadequate vascular pattern (type B2) for DFFMT. Therefore, we had total 80 patients received the 1^st^ stage free gracilis-adductor DFFMT and the 2^nd^ stage single gracilis FFMT; so called **“Triple FFMT”**; for complete total avulsion BPI. There were 70 males and 10 females. The average age was 33.5 years old. The shoulder function was reconstructed by neurotization. The 1^st^ DFFMT reconstructive procedures were performed in an average 3.5 months (from 2 months to 6 months) after trauma. The 2^nd^ gracilis FFMT was performed in an average 3 months (2 to 4.5 months) after the 1^st^ FFMT surgery. The 1^st^ DFFMT was serving as elbow flexor and finger/wrist extensor, while the second gracilis FFMT was serving as finger flexor in the 2^nd^ stage surgery. The average follow up was 7.5 years.

## Results

The primary flap success rate (including 1^st^ & 2^nd^ stage surgeries) was 97.5% (156/160 flaps), with 4 cases requiring reopen surgery due to venous thrombosis. 87.5% (70/80) achieved M3 elbow flexion and M3 finger/wrist extension, and 65% (52/80) obtained M3 hand grip in 1 year follow up. In 3 years follow up, 90 % (72/80) could have M4 elbow function and M4 finger/wrist extension, but only 70% (56/80) had M4 hand grip. The reasons for failure are: flap re-open, use of previously used nerve, tendon adhesion, lack of adequate rehabilitation, and inadequate skin coverage. The most significant recovery of motor function happened during the 9 months to 24 months after the triple FFMT surgery.

## Conclusion

We concluded that triple FFMT for reconstruction of total avulsion type BPI is a worthwhile technique. This technique could offer better finger/wrist extension function than DFFMT, by adding one more FFMT in the 1^st^ stage surgery.

**Figure 1:**
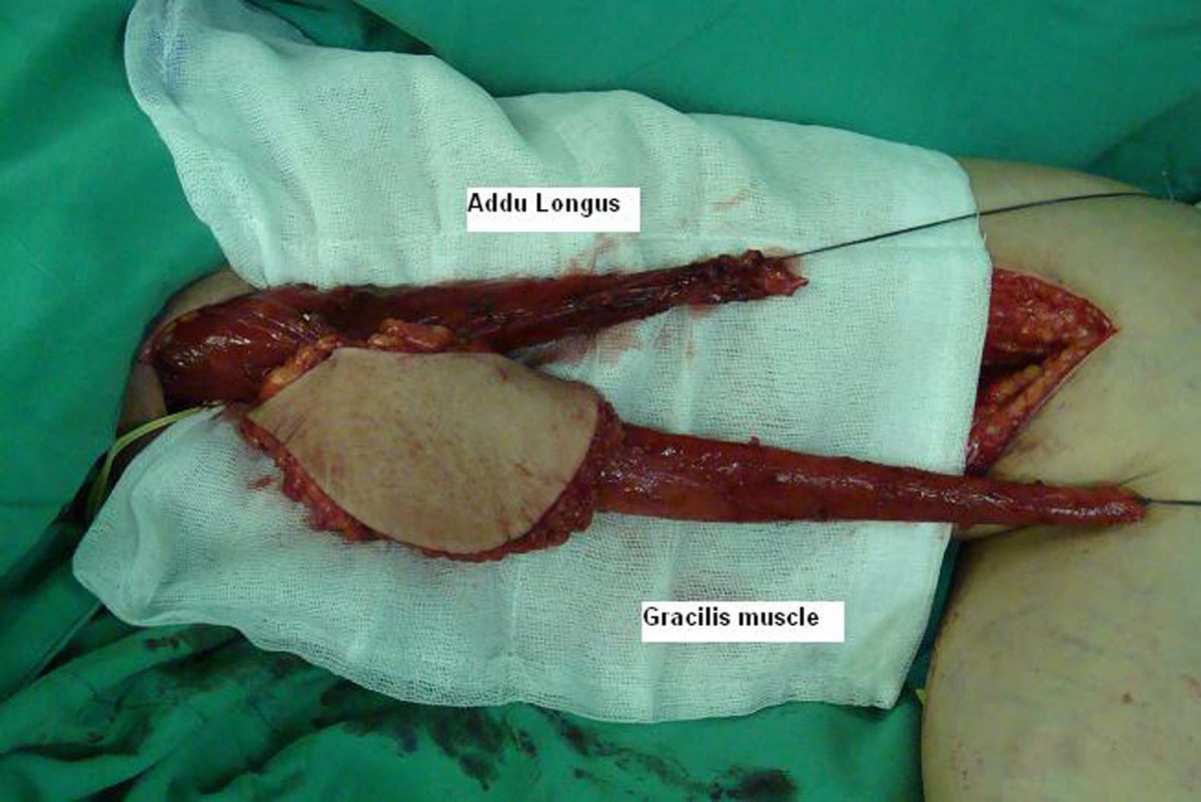
The combined Adductor longus-Gracilis FFMT

